# Tissue-Engineering Strategies to Repair Chondral and Osteochondral Tissue in Osteoarthritis: Use of Mesenchymal Stem Cells

**DOI:** 10.1007/s11926-014-0452-5

**Published:** 2014-09-03

**Authors:** Susanne Grässel, Julia Lorenz

**Affiliations:** 1 Experimental Orthopedics, Centre for Medical Biotechnology, BioPark 1, Department of Orthopedic Surgery, University Hospital Regensburg, Regensburg, Germany; 2 Orthopedic Surgery, Exp. Orthopedics, University of Regensburg, ZMB / BioPark 1, Josef-Engert-Str. 9, 93053 Regensburg, Germany

**Keywords:** Osteoarthritis, Trauma, Cartilage, Focal defect, Clinical, Preclinical, Animal model, Ovine, Porcine, Equine, Chondral, Osteochondral, Hyaline, Mesenchymal stem cells, Platelet-rich plasma, Bone-marrow concentrate, Tissue engineering, Collagen, Fibrin gel

## Abstract

Focal chondral or osteochondral lesions can be painful and disabling because they have insufficient intrinsic repair potential, and constitute one of the major extrinsic risk factors for osteoarthritis (OA). Attention has, therefore, been paid to regenerative therapeutic procedures for the early treatment of cartilaginous defects. Current treatments for OA are not regenerative and have little effect on the progressive degeneration of joint tissue. One major reason for this underrepresentation of regenerative therapy is that approaches to treating OA with cell-based strategies have to take into consideration the larger sizes of the defects, as compared with isolated focal articular-cartilage defects, and the underlying disease process. Here, we review current treatment strategies using mesenchymal stem cells (MSCs) for chondral and osteochondral tissue repair in trauma and OA-affected joints. We discuss tissue-engineering approaches, in preclinical large-animal models and clinical studies in humans, which use crude bone-marrow aspirates and MSCs from different tissue sources in combination with bioactive agents and materials.

## Introduction

Focal chondral or osteochondral lesions can be painful and disabling, and predispose patients to osteoarthritis (OA). For long-term repair and regeneration of these defects, cells alone or in combination with biomaterials are implanted at the site of injury. However, not much attention has been paid to microenvironmental effects of the neighboring cartilage and subchondral bone. This is particularly evident in diseases affecting diarthrodial joints, including OA, which is an age-related and/or trauma-induced multi-factorial, slowly progressing, and primarily non-inflammatory degenerative disorder of the synovial joints culminating in the irreversible destruction of the articular cartilage. As well as metabolic imbalance, activation of the whole endochondral-ossification program, starting with cell proliferation through articular-chondrocyte hypertrophy and apoptosis, has been identified as an important determinant of OA progression [1–4].

Articular cartilage lesions greater than 5 mm^2^ do not heal spontaneously [5], and for therapy it must be kept in mind that cartilage defects are multifactorial and site-specific and thus need both a clear analysis of the underlying pathology and individualized therapy. Chondral or osteochondral lesions of any type are found in 61 % of patients with joint pain and are the most prevalent indication for surgical cartilage repair. The incidence of severe International Cartilage Repair Society (ICRS) grade III lesions and of full chondral ICRS grade IV defects in knee joints are approximately 40 % and 19 %, respectively [6]. If left untreated they lead, after a long asymptomatic interval, to full clinical OA. It has been suggested that cartilage repair surgery may be most suitable for patients younger than 40–50 years. Attention, therefore, has been paid to therapeutic procedures for the early treatment of cartilaginous defects. The advantage of local defects is that they are contained within healthy cartilage and bone, and it is likely that delivery of specific growth factors and other chondroprotective factors to defect sites will support cartilage healing. Early treatment of cartilaginous lesions could indeed be crucial to slowing down the chronic development of OA. The major challenges in regenerative medicine for cartilage are restoration of a biomechanically competent extracellular matrix (ECM) and intimate integration of this newly synthesized matrix within the resident tissue. To address this specific challenge, autologous chondrocyte implantation (ACI) was developed and has prepared the way for novel cell-based therapy and biomaterial-assisted cartilage engineering [7].

Current treatments for OA are not regenerative and have little effect on the progressive degeneration of joint tissue. Clinical interventions are primarily symptomatic, with a focus on pain reduction and control of inflammation using non-steroidal anti-inflammatory drugs and, ultimately, total joint replacement [8, 9]. One major reason for this underrepresentation of regenerative therapy is that approaches for treating OA with cell-based strategies have to take into consideration both the larger sizes of the defects and the underlying disease process. Fragile neocartilage constructs produced by implanted or injected mesenchymal stem cells (MSCs) or chondrocytes may undergo rapid degradation when situated in inflamed or diseased joints. Therefore the underlying pathology must be brought effectively under control, because otherwise any cell-based treatment strategy of OA is unlikely to be successful long-term. This knowledge implies that cartilage repair lacks a one-for-all therapy.

**Fig. 1:**
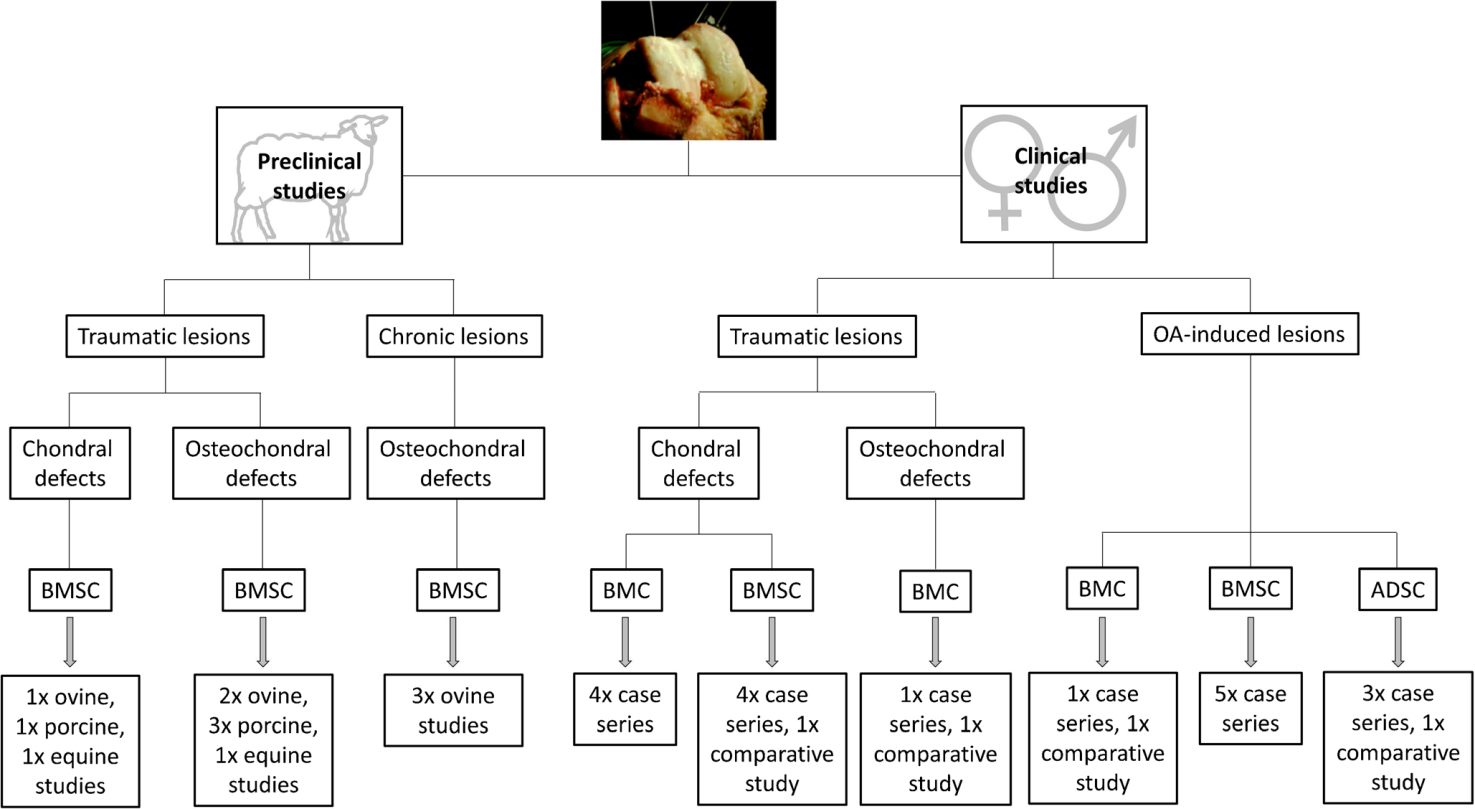
Overview of preclinical and clinical studies of treatment of traumatic and osteoarthritis-induced defects with mesenchymal stem cells (MSCs) discussed in this review. ADSC = adipose-tissue-derived stem cells, BMC = bone-marrow concentrate, BMSC = bone-marrow-derived mesenchymal stem cells

Here, we present up-to-date treatment strategies using MSCs for chondral and osteochondral tissue repair in post-traumatic and OA-affected joints. We have reviewed current literature for tissue-engineering approaches in preclinical large-animal models and clinical studies of humans using crude bone-marrow concentrate (BMC) and MSCs from different tissue sources in combination with other agents. Figure 1 gives an overview of preclinical and clinical studies referred to in this review.

### Clinical-Application Techniques for Autologous BMC and/or MSC

In principal, two strategies exist for the clinical application of cultured and expanded cells (autologous chondrocytes or MSCs) or cell suspensions (autologous bone-marrow concentrates or whole blood): surgical treatment and injection. Surgical delivery of cells includes transplantation or implantation of cells into the chondral and osteochondral defects. The advantage of this approach is the direct and targeted application on the lesion site; the disadvantage is the invasiveness of the approach, which requires opening of the joint cavity. Intraarticular injections are less invasive, which makes application easier; however, there is no precise way of delivering the cells to the defect site. Depending on the application method, the suspension can be injected directly into the diseased tissue, where the cells eventually populate the target site and stimulate repair via autocrine or paracrine pathways. With respect to human therapy, injection of whole blood or bone marrow, or concentrates thereof, can usually be performed in the operating theatre without major regulatory obstacles. The administration of cell preparations expanded ex vivo requires strict compliance with good medical practice (GMP) and nation-specific requirements.

Bone-marrow concentrate has attracted much interest from orthopedic surgeons as a third-generation intra-articular orthobiological injectable therapy for cartilage disease. Bone-marrow concentrate contains MSC, hematopoietic stem cells, platelets (containing growth factors), and cytokines. The anti-inflammatory and immunomodulatory properties of bone-marrow-derived mesenchymal stem cells (BMSC) or adipose-derived stem cells (ADSC) can facilitate regeneration of tissue. Although marrow suspensions are believed to contain a higher percentage of stromal cells than peripheral blood, both are crude mixtures with low overall abundance of MSCs and undefined mixtures of proteins. The MSC density can be increased by centrifugation of whole blood or marrow, recovery from cell filters, clotting, or other concentration methods. These methods are relatively simple, making them appealing for single-step usage procedures in the operating theatre, and, therefore, are not associated with major regulatory obstacles.

Alternatively cells, and in particular MSCs, can be harvested from blood, bone marrow, or, after digestion, other tissues via their adherence to plastic surfaces. Such cell populations are usually maintained in tissue culture for amplification under controlled GMP conditions using autologous serum. This method offers the opportunity for close monitoring of the cells regarding safety and quality aspects, and for further cell selection. However, this clinical approach has the greatest regulatory obstacles in the specific requirements of each country and continent (European Medicines Agency and U.S. Food and Drug Administration). The use of unprocessed and ex-vivo-processed cells might be enhanced and supplemented by the use of biomaterial scaffolds, soluble growth factors, nucleic acids, or mechanical stimulation. Additional specific regulatory requirements must be met for the use of each of these supplements and for their combined usage, indicating the complexity of such approaches from a biological, medical, and regulatory perspective (reviewed in Refs. [10, 11•]).

### Treatment of Chondral and/or Osteochondral Lesions with MSC in Preclinical Studies Using Large-Animal Models

The average human articular-cartilage thickness is 2.2–2.5 mm [12] and the average defect size is approximately 550 mm^3^ [6, 13], with 95 % of defects involving cartilage only and not affecting subchondral bone [6, 14]. For testing cell-based treatments, a perfect animal model should mimic as precisely as possible the human defect and articular-cartilage morphology. A variety of animal models for cartilage lesions are described in literature, ranging from murine, laprine, canine, ovine, porcine, and caprine to equine models (reviewed in Ref. [14]). Small-animal models, for example mouse and rabbit, are often used as proof-of-concept, but articular cartilage is thinner and defects are much smaller compared with humans; in addition, most defects cannot be set without penetrating the subchondral bone plate. Therefore, especially for preclinical studies, large animals including swine, sheep, or horse are more appropriate for modelling human articular-cartilage defects and testing the regenerative capacity of cell-based treatments. The following section presents a comprehensive overview of published studies on MSC-based treatment for regenerating localized chondral and osteochondral defects in ovine, porcine, and equine animal models. Table 1 summarizes conditions and treatment outcomes.

**Table 1:** Preclinical trials using BMSC for chondral and osteochondral repair in ovine, porcine, and equine animal models

Reference	BMSC	Experimental groups (pre-incubation, matrix, scaffold, and/or growth factors)	No. of animals	Follow-up (m)	Defect and defect site	Defect size	Results (% of normal tissue)
Mrugala et al., 2008 [15]	Ovine, p1, 10^7^	1: Fibrin glue 2: Chitosan + fibrin glue 3: BMSC + fibrin glue 4: BMSC + chitosan + fibrin glue 5: BMSC + chitosan + TGF-β3 + fibrin glue 6: Chitosan + TGF-β3 + fibrin glue	3	2	Chondral partial thickness in internal groove of patella	Diameter 4 mm	No scoring was performed. When BMSC were combined with chitosan and TGF-β3, repaired tissue had chondrocytes like rounded cells surrounded by hyaline-cartilage-like tissue.
Zscharnack et al., 2010 [16]	Ovine, p1, 7.2 × 10^5^	1: Cells in collagen I gel, 2 weeks in chondrogenic medium (TGF-β3) 2: Cells in collagen I gel, 2 weeks in expansion medium 3: Collagen I gel 4: Non-treated	10	6	Osteochondral in medial femoral condyle	Diameter 7 mm, 77 mm^3^	1: 73 % ^a^ 2: 47 % 3: 28 % 4: 40 % (O’Driscoll score)
Marquass et al., 2011 [17•]	Ovine, p1, 7 × 10^5^	1: Cells in collagen I gel, 2 weeks in chondrogenic medium (TGF-β3) 2: Cells in collagen I gel, 2 weeks in expansion medium 3: Chondrocytes in collagen I gel, 4 weeks in expansion medium 4: Non-treated	9	12	Osteochondral in medial femoral condyle	Diameter 7 mm, 77 mm^3^	1: 76 % ^a^ 2: 44 % 3: 56 % 4: 45 % (O’Driscoll score)
Marquass et al., 2010 [18]	Ovine, p1, 4 × 10^5^ chondral phase, 10^6^ osseous phase	1: Triphase implant: cells in collagen I gel, 2 weeks in chondrogenic medium (TGF-β3) (chondral phase) / plasma / cells seeded on β-TCP implants + EDTA plasma + 0.1 mol L^−1^ CaCl_2,_ 2 weeks in expansion medium (osseus phase) 2: OATS	5	6 and 12	Osteochondral in medial femoral condyle	Diameter 6.4 mm, 386 mm^3^	1: 56 % 2: 68 % (O’Driscoll score after 12 months)
Guo et al., 2004 [19]	Ovine, p3, 3 × 10^7^	1: Cells were seeded without pre-differentiation on β-TCP implants, cell attachment for 30 h 2: β-TCP implants without cells 3: Untreated	8	3 and 6	Osteochondral in medial femoral condyle	Diameter 8 mm, 201 mm^3^	1: 75 %^a^ / 87 %^a^ 2: 42 % / 19 % 3: 21 % / 9 % (O’Driscoll score / GAG content after 6 months)
Frosch et al., 2006 [20]	Ovine, p2	1: Cell-coated titanium implant, 3–4 weeks migration in implants 2: Cell-free titanium implant 3: Untreated	–	–	Osteochondral in medial femoral condyle	Diameter 7.3 mm, 209 mm^3^	1: 44 % 2: 28 % 3: 14 % (Wakitani score)
Steck et al., 2009 [21]	Porcine, p3, 2 × 10^6^	1: Undifferentiated BMSC in fibrin glue, defect covered with collagen I and III matrix 2: In-vitro differentiation in fibrin, 2 months with chondrogenic medium (+TGF-β3, +BMP-2)	6	2	Chondral full thickness in medial trochlear facet	Diameter 5.4 mm, 34–46 mm^3^	In vivo, significantly lower MMP-13 and collagen X level related to collagen II expression compared with in vitro
Zhou et al., 2006 [22]	Porcine, p2, 1.5 × 10^7^	1: Chondrogenically inducted BMSC seeded on PGA–PLA construct, 1 week in expansion medium (+TGF-β1) 2: BMSC seeded on PGA–PLA construct, 1 week in expansion medium (−TGF-β1) 3: PGA–PLA construct alone 4: Untreated	6 10	3 6	Osteochondral in femur trochlea	Diameter 8 mm, 302 mm^3^	1: 85 % / 80 % / 93 % 2: 67 % / 63 % / 78 % 3: 24 % 4: 15 % (mod. Wakitani and Pineda score / compressive moduli / GAG content)
Ho et al., 2010 [23]	Porcine, p1, 5 × 10^6^ (PCL), 2 × 10^6^ (PCL–TCP)	1: Undifferentiated BMSC via fibrin in PCL and PCL–TCP, covered with PCL–collagen electrospun mesh 2: Undifferentiated BMSC via fibrin in PCL and PCL–TCP 3: Fibrin in PCL and PCL–TCP, covered with PCL–collagen electrospun mesh	6	6	Osteochondral in medial condyle and patella groove	Diameter 8 mm, 402 mm^3^	Medial condyle: 1: 65 % / 70 % 2: 65 % / 68 % 3: 58 % / 42 % (mod. O’Driscoll score / Young’s modulus)
Chang et al., 2011 [24•]	Porcine, p4, 10^6^	1: Cells in collagen I gel, 2 weeks in chondrogenic medium (+TGF-β1), periosteal patch from the tibia 2: Cells in collagen I gel, 2 weeks in chondrogenic medium (−TGF-β1), periosteal patch from the tibia 3: Collagen I gel, periosteal patch 4: Non-treated	1: 10 2: 6	6	Osteochondral in medial femoral condyle	Diameter 6.5 mm, 100 mm^3^	1: 44 % / 51 % 2: 67 % / 51 % 3: 48 % / 56 % 4: 41 % / 50 % (Pineda score / compressive stiffness)
Wilke et al., 2007 [25]	Equine, p2/3, 1.2 × 10^7^	1: Undifferentiated BMSC in autologous fibrin 2: Autologous fibrin without cells	6	8	Chondral full thickness in femoral patella	Diameter 15 mm, 309–353 mm^3^	1: 57 % / 54 % / 184 % / 38 % 2: 52 % / 51 % / 197 % / 55 % (score / collagen II / DNA / GAG content)
Seo et al., 2013 [26•]	Equine, p1, MSC 5 × 10^6^, Ch 5 × 10^4^	1: Chondrogenic layer: Ch differentiated for 10 days + BMSC + PRP (source for TGF-β1) seeded on acidic GT, osteogenic layer: BMSC + BMP-2 seeded on basic GT sponge (biphasic) 2: Bilayer GT sponge without cells and growth factors	6	4	Osteochondral in lateral trochlear ridge of talus	Diameter 4.5 mm, 159 mm^3^	1: 55 % 2: 39 % (Niederauer score)

BMP = bone morphogenetic protein, BMSC = bone-marrow-derived mesenchymal stem cells, Ch = chondrogenically differentiated BMSC, GAG = glycosaminoglycan, GT = bilayer gelatin–β tricalcium phosphate, m = months, MMP = matrix metalloproteinase, OATS = osteochondral autografting, p = passage, PCL = polycaprolactone, PGA–PLA = polyglycolic acid–polylactic acid, PRP = platelet-rich plasma, TGF = transforming growth factor, TCP = tricalcium phosphate osseous. ^a^ = Group 1 is significantly different from other groups

#### Ovine Models

Sheep are commonly used as animal models for chondral and osteochondral defects, because these animals are readily available, easy to handle, and relatively inexpensive. In addition, the anatomy of the knee joint is similar to that of humans and “second look” arthroscopy is possible. Furthermore, the ovine medial femoral condyle cartilage thickness of 0.4–1.7 mm [12, 27]—although thinner than that of humans—enables production of partial and full-thickness defects that do not penetrate the subchondral bone plate [14]. In osteochondral defects the bone marrow is opened, and one likely situation is that endogenous BMSCs migrate from the bone marrow into the defect, thus contributing to the repair tissue in a manner comparable to the BMSC implant. This setting makes it impossible to determine whether implanted cells or endogenous cells are responsible for defect healing. To circumvent this situation, one ovine study conducted by Mrugala et al. [15] implanted autologous ovine BMSCs into a partial-thickness-cartilage-defect model. BMSCs in passage 1 were mixed with blood and/or chitosan scaffold and/or TGF-β3, and the clots generated were filled into cartilage defects set in the internal groove of the patella. The authors observed that BMSCs or the scaffold alone had poor ability to repair these defects, whereas BMSC in the presence of TGF-β3 and chitosan-based-scaffold-generated tissue was positive for aggrecan and collagen II, indicating hyaline-like repair tissue. After two months the defects were filled with this cartilage-like tissue; this tissue was only moderately integrated with the surrounding cartilage, indicating that investigations at later time points are required to observe full integration of newly formed tissue occurring after several months (Table 1).

For BMSC treatment of ovine osteochondral defects, five studies have been reported. A novel, promising approach seems to be the application of BMSCs that have been pre-differentiated ex vivo to the chondrogenic stage; this strategy was tested in three studies by the same group using comparable experimental setups [16, 17•, 18]. A unique aspect of these three studies was that the treatment was performed six weeks after defect setting, whereas in all other large-animal studies defects were treated directly after setting. Delaying treatment after defect setting creates a clinically relevant situation that resembles chronic post-traumatic human OA. In the first study by Zscharnack et al. [16], pre-differentiated BMSCs achieved better regenerative results after six months, as measured by gross appearance and O’Driscoll [28] and ICRS [29] scores with respect to collagen II content and hyaline-cartilage appearance, than undifferentiated BMSCs, collagen I gels alone, and untreated controls. However, integration of newly formed cartilage-like tissue was incomplete at six months, and the important question of whether the BMSCs had adopted a stable chondrogenic phenotype, without further progression to hypertrophy at time points beyond six months, was not addressed. In the second study, Marquass et al. [17•] compared the regenerative capacity of ex-vivo-pre-differentiated BMSCs with differentiated autologous articular chondrocytes after 12 months, and found higher O’Driscoll [28] and ICRS scores with pre-differentiated BMSCs in collagen I gels than with chondrocytes in MACT gels, undifferentiated BMSCs in gels, and untreated controls. Furthermore, repair tissue generated by pre-differentiated BMSCs had no sign of degradation within one year. In this line, the approach was enhanced by embedding pre-differentiated BMSCs into triphasic constructs [18]. These triphasic constructs were compared with standard osteochondral autografting (OATS), again using an ovine osteochondral-defect model. At six months no significant difference in histological scores between the two groups was detected. At this time point the triphasic-construct group had superior cartilage bonding, whereas at 12 months the OATS group had superior cartilage-matrix morphology. Because of an observed sinking of the triphasic constructs, and a high variability in the quality of the repaired tissue, the authors did not believe the triphasic construct superior to OATS. A study from Guo et al. [19] used BMSCs without pre-differentiation seeded on β-tricalcium phosphate (TCP) implants for treatment of osteochondral defects. After six months, hyaline-like tissue covered the surfaces of the defects and a perfect interface between engineered tissue, the adjacent cartilage, and underlying bone was observed, whereas in control groups (β-TCP scaffold and untreated) the defects were clearly visible and incompletely repaired. The authors concluded that undifferentiated MSCs on β-TCP scaffolds have promising potential for clinical application. The last ovine study analyzed the regenerative capacity of undifferentiated BMSCs seeded in titanium implants into large osteochondral defects. After six months, Frosch et al. [20] found that 50 % of BMSC-coated implants were osseo-integrated, with complete regeneration of subchondral bone; the regenerated cartilage contained collagen I and collagen II, indicating hyaline-like-cartilage regeneration. In contrast, the other 50 % of the BMSC-coated implants, the cell-free implants, and the non-treated controls had incomplete healing, osseo-integration failure, and formation of fibrocartilage instead of hyaline cartilage (Table 1).

#### Porcine Models

Pigs are not commonly used for cartilage-defect research, because of handling difficulties and logistical requirements regarding housing. A point in favor of using pigs is that the articular cartilage thickness of 1.5–2.0 mm [12, 30, 31] is more similar to humans than that of ovine cartilage and enables easier production of partial and full-thickness defects without penetrating the subchondral bone plate [14], better reflecting human articular-cartilage defects.

Proof of a stable in-vivo cartilaginous-tissue phenotype of implanted BMSCs is the focus of a study by Steck et al. [21]. The group analyzed expression of collagens II and X and of MMP-13 to compare the performance of BMSCs pre-differentiated in vitro to a chondrogenic stage versus undifferentiated BMSCs when implanted into minipigs with acute, full-thickness cartilage defects on the trochlear ridge of the femur. Defects were filled with the undifferentiated BMSCs and sealed with a collagen I and III matrix and commercial fibrin glue. Follow-up times for assessment of morphological and molecular tissue aspects were up to eight weeks. The data revealed that, in comparison to the pre-differentiated BMSCs, the application of undifferentiated BMSC in the orthotopic environment resulted in lower COL10A1/COL2A1 and MMP13/COL2A1 mRNA ratios. The reason for the discrepancy between in-vitro and in-vivo results is poorly understood, and the authors speculated that unknown signaling molecules and biomechanical stimuli derived from neighboring cartilage and/or bone tissue may have an important effect. On the basis of their data, they regarded application of BMSCs pre-differentiated ex vivo as unfavorable for cartilage repair until better in-vitro induction procedures for chondrogenesis become available. In addition, to obtain a repair tissue comparable to healthy cartilage, a study duration of eight weeks is too short.

The regenerative capacity of BMSCs in porcine osteochondral defects was reported in three studies. Zhou et al. [22] analyzed the repair capacity of pre-differentiated and undifferentiated BMSCs in biodegradable PGA–PLA scaffolds. After six months, both groups with cells had better reparative results regarding gross appearance and histology than were obtained for the group with scaffold alone and the untreated group. Repair tissue of pre-differentiated BMSCs had almost normal cartilage and subchondral-bone-like properties. Concerning the question of if and for how long implanted BMSCs remain in the defect, a study revealed that GFP-labeled implanted BMSCs are located in the regenerated cartilage-like tissue and subchondral bone even after seven months of follow-up. In a carefully designed and evaluated study, Ho et al. [23] used undifferentiated BMSCs embedded with fibrin in polycaprolactone (PCL) and PCL–TCP scaffolds and covered the defect with PCL–collagen electrospun mesh. In contrast with the findings of Zhou et al., defects were set in weight-bearing areas (medial condyle and patellar groove) of the joint. After six months, BMSCs and PCL–collagen electrospun mesh had a positive effect on morphological outcomes of cartilage-like repair tissue. Notably, healing was inferior at the patellar groove compared with the medial condyle, and this was attributed to site-specific biomechanical features.

Chang et al. [24•] obtained better histological Pineda scores [32] with undifferentiated BMSCs compared with differentiated BMSCs, collagen I matrix alone, or untreated defects in minipigs after six months. However, the quality of the regenerated tissue was inferior and did not differ significantly between experimental and control groups. In contrast with the two previously described studies, osteochondral defects were smaller—which may explain the high degree of spontaneous healing of non-treated defects—when BMSCs were used at higher passages without scaffolds inserted into the defect (Table 1).

#### Equine Models

The horse is the largest model available and, having an average medial-femoral-condyle-cartilage thickness of 1.75–2 mm [33], it has even more similarity to human cartilage in thickness than other large-animal models. “Second look” arthroscopy is possible, and partial or full-thickness cartilage defects without penetration of the subchondral bone plate can be set. However, the horse is a companion animal and thus ethical aspects are an important factor. Furthermore, horses are not bred specifically for biomedical research, handling is difficult, and housing is very expensive [14].

In Wilke et al. [25], 15 mm full-thickness cartilage defects were set in the femoral patella and filled with undifferentiated BMSCs embedded in autologous fibrin. A second-look arthroscopy and biopsy were obtained for each animal after 30 days and animals were euthanized after eight months. Short-term assessment revealed a significant improvement of arthroscopy scores and increased collagen II-containing fibrous tissue in defects treated with BMSCs compared with those treated with fibrin matrix alone. However, long-term assessment revealed no difference in BMSC-treated defects compared with those treated with fibrin matrix alone regarding GAG and/or collagen II content and matrix biochemical assays. For both groups, the repair tissue differed markedly from normal cartilage in cartilage-quality scores and biochemical properties. In this study defect healing might be affected by compromised motion because, after recovery from anesthesia, the horses were housed without exercise for five weeks and then with a daily exercise regimen of a 5 min walk for a further seven weeks.

Seo et al. [26•] analyzed the repair capacity of BMSCs loaded on biphasic sponge scaffolds and implanted in osteochondral defects. The chondrogenic layer consists of a acidic gelatin–β-tricalcium phosphate (GT) sponge loaded with platelet-rich plasma, BMSCs, and chondrogenically differentiated BMSCs (without TGF-β), whereas the osteogenic layer is composed of a basic GT sponge loaded with BMP-2 and BMSCs. After four months, the test group had significantly higher radiographic, QCT, macroscopic, and histological scores than the control group with biphasic sponge and without cells or growth factors (Table 1).

#### Comparison to Human Situation

Despite the fact that 95 % of human defects are of chondral and not osteochondral nature [6, 14], only a few pre-clinical studies [15, 21, 25] have created isolated chondral defects to analyze the cartilage-regeneration capacity of BMSCs. Furthermore, most studies suffer from too-short follow-up times—usually less than 12 months—which do not enable satisfactory analysis of repair tissue with respect to stability of the chondrogenic phenotype, hyaline-like appearance, defect-site integration, and biomechanical characteristics. For osteochondral defects, all large-animal studies, irrespective of the animal model, reported improvement in regeneration when BMSCs were used for defect repair [16, 17•, 20, 22, 23, 24•, 25]. A beneficial effect of an additional ex-vivo pre-differentiation phase of MSCs is a matter of debate on the basis of current knowledge [16, 17•, 21, 22].

Several aspects remain to be addressed in future studies, including the effect of animal age on the extent of repair of chondral defects at weight-bearing areas. Importantly, only the three ovine studies from the Leipzig group featured a study design with a six-week gap between defect setting and implantation of BMSCs, thereby inducing an early OA stage. In addition, thus far only MSCs isolated from bone marrow have been used. Other MSC sources, for example adipose tissue, might provide many advantages because of easier cell preparation, a higher percentage of MSCs compared with differentiated cells, and better availability. No study has been performed using only BMCs to study the cartilage-regeneration capacity of crude cell suspensions including those used frequently for human treatment (see below). Most importantly, in all studies (except the one from the Richter group [21]) no specific marker for hypertrophic differentiation of the BMSCs was evaluated and therefore no conclusion can be drawn regarding the phenotypic stability of the implanted cells. Furthermore, to find out under which conditions regenerated tissue best reflects normal tissue-like properties, the overall comparability between the study procedures must be improved with respect to follow-up times, scoring systems, biomechanical measurements, and biochemical properties.

### Human Clinical Studies Using Autologous MSC and BMC for Treatment of Chondral and Osteochondral Lesions: Promising Tissue-Engineering Approaches

Cartilage defects that arise from an underlying disease process (for example OA) are distinct from focal cartilage lesions that result from acute injury or osteochondrosis dissecans, and this difference must always be taken into consideration. Specifically, acute cartilage injury and osteochondrosis dissecans often occur in otherwise healthy joints; the patient might be young, and probably only the focal defect will require localized treatment. In contrast, patients with OA are likely to be elderly, and often the entire articulating surface will require treatment. Repair of lesions may provide symptomatic relief and delay the progression of OA symptoms, but without effective treatment of the underlying disease any improvement is likely to be short-lived [34]. Thus, common to all types of cell-based therapy is that they are not recommended for treatment of expanded, arthritic cartilage lesions and kissing lesions. Rather, they are recommended for treatment of local, full-thickness chondral and osteochondral defects, ideally with an intact cartilage interface [35].

#### Trauma-Induced Chondral and Osteochondral Defects

##### Microfracturing

If an articular cartilage lesion below 1–4 cm^2^ is confirmed, the first choice of treatment is often microfracturing (MFX), the penetration of the subchondral bone plate by creating small holes. Bleeding from the subchondral bone spaces yields a blood clot (often called superclot), which is believed capable of stimulating attraction, proliferation, and chondrogenic differentiation of MSCs arising from the bone marrow. The advantage of a marrow-stimulating technique is that several of the main objectives of cartilage repair are fulfilled: it is an easy, simple, minimally invasive, low-morbidity, and single-stage procedure, and is a cost-effective technology with few associated complications and a high capacity for creation of durable cartilage-repair tissue that can delay the time to joint replacement [36, 37]. However, clinical results are age-dependent. Marrow stimulation results in improved function and reduced pain for up to 75 % of young patients after five years and up to 80 % of patients after an average of 11 years of follow-up [38, 39]. Repair tissue is mostly fibrocartilage or a hybrid of hyaline and fibrous cartilage, of inferior quality and mechanical stability compared with the original cartilage matrix. Formation of an insufficient cartilage ECM might be promoted by an inadequate microenvironment of the superclot, because its composition is poorly defined. Other limitations are that only partial defect filling is achieved, because of shrinking of the superclot and low incidence of MSCs in the bone-marrow fraction (Reviewed in [37, 40]).

##### Application of BMC to Trauma-Induced Defects

MSC can be used as a cell suspension expanded by culture or from bone-marrow concentrate (BMC). However, these products differ profoundly in composition. The advantage of using BMC over cultured MSCs is that the cells are not passaged, but the disadvantage is that BMC gives a more heterogeneous composition compared with expanded MSC [41•]. Studies have been reported in which BMC, usually combined with other regenerative techniques, is applied to trauma-induced human-cartilage defects. The group of Gigante published three case series in which they treated five patients with isolated femoral condyle lesions by combining BMC with the autologous matrix-induced chondrogenesis (AMIC) technique [42]. In a second study, they described a novel arthroscopy technique that combines MFX, autologous BMC, and a protective collagen-based scaffold [43]. The objective of both procedures is to augment the original single-stage procedure with BMC and to increase defect filling and the rate of hyaline-like-cartilage regeneration. The procedure combining MFX, BMC, and a protective collagen scaffold is inexpensive and reproducible and has obtained regeneration of hyaline-like cartilage. A third study from this group reported on a combination of MFX, BMC, and resorbable polyglycolic acid–hyaluronic acid (PGA–HA) membranes. Nine patients with focal lesions of the condylar articular cartilage were consecutively treated with arthroscopic PGA–HA-covered MFX and BMC. Macroscopic assessment of cartilage after 12 months revealed that one repair attempt appeared normal, three almost normal, and one abnormal. Histological analysis revealed hyaline-like-cartilage-repair-tissue formation in one case and MRI at 8–12 months follow-up revealed complete defect filling [44•]. A comparable approach to treating osteochondral knee and talar dome lesions is reported by the Giannini group, who combined BMC and HA-scaffolds (or collagen powder) with platelet-rich fibrin (PRF) gel in a one-step repair technique and treated 48 patients, with an average follow-up of 24–35 months. Histological evaluation revealed regenerated tissue with a variety of degrees of remodeling irrespective of biomaterial used, although none had entirely hyaline cartilage. These data suggest the one-step technique is a possible alternative for cartilage repair, obtaining improved functional scores and overcoming the disadvantages of previous techniques [45]. In this line, a recent case-series study reported treatment of large cartilage lesions of up to 12 cm^2^ (ICRS grade III–IV) with BMC, resulting in significant improvement in all clinical scores for 52 out of 54 patients [46]. In a study comparing BMC, open autologous chondrocyte implantation (ACI), and ACI arthroscopy, clinical improvement was similar in all three groups [47] (Table 2).

**Table 2:** Clinical trials using BMC and MSC for chondral and osteochondral repair

Reference	Study type	Indication and defect size	No. of patients	Follow-up	Cells and suspensions	Results
Gigante et al., 2011 (Italy) [42]	Case series	Cartilage lesion of medial femoral condyle	5 (mean age 43.4 y)	12 m	BMC + collagen membrane (AMIC)	Nearly normal arthroscopic appearance of implants; mean histological score (ICRS II); hyaline-like matrix was found in 1 patient; a mixture of hyaline and fibrocartilage was found in 1 patient; fibrocartilage was found in 3 patients.
Gigante et al., 2012 (Italy)[43]	Case report	Cartilage lesion of medial femoral condyle 3 cm^2^	1 (age 37 y)	24 m	3–5 mL BMC + MFX + collagen membrane (AMIC)	No pain after 6 m; MRI at 12 m revealed good defect filling and no bone edema; at 24 m patient was still asymptomatic
Enea et al., 2013 (Italy) [44•]	Case series	Focal condylar articular knee lesions	9	22 ± 2 m	MFX + PGA–HA matrix + BMC	Eight patients had significant improvement in mean IKDC subjective score, Lysholm score, VAS, and median Tegner score up to 22 m; one cartilage repair appeared normal, three almost normal, one appeared abnormal, and one appeared hyaline-cartilage-like. MRI at 8–12 m revealed complete defect filling
Giannini et al., 2009 (Italy) [45]	Case series	Focal osteochondral lesions of the talar dome. Size: 2.07 ± 0.48 cm^2^ Depth: 4.0 ± 0.9 mm	48 (age 28.5 ± 9.5 y)	24–35 m	2 mL BMC + collagen powder (or HA membrane) + 1 mL PRF	Increase of clinical score (AOFAS, MRI revealed newly formed tissue with hyaline-like characteristics but hypertrophic appearance; close integration with surrounding cartilage
Giannini et al., 2010 (Italy) [47]	Comparative study	Focal osteochondral monolateral lesions of the talar dome. Size: 2.18–0.5 cm^2^ Depth: 4.0–0.9 mm	81 total (age 30 ± 8 y): 10 (ACI); 46 ACI arthroscopic; 25 BMC	36 m	ACI open; ACI arthroscopic injection; 2 mL BMC + collagen powder (or HA membrane) + 1 mL PRF	Clinical score (AOFAS) improvement in all 3 groups; MOCART (MRI) scoring system revealed nearly complete integration of regenerated tissue with surrounding cartilage in 76 % of patients; in 5 cases hypertrophy of regenerated tissue; histology revealed hyaline-like cartilage and tissue remodeling
Skowronski et al., 2013 (Poland) [46]	Case series	ICRS grade III or IV cartilage lesions. Size: 4–12 cm^2^	54	1 and 5 y	BMC + collagen membrane	KOOS and Lysholm functional scales and VAS and KOOS pain scales increased in 52 patients. No MRI data, no histology data
Wakitani et al., 2004 (Japan) [48]	Case report	Full-thickness articular-cartilage defects in patellae	2 (age 26 y and 44 y)	4 and 5 y	Transplantation of expanded BMSC in collagen gel + periosteum	Improvement of clinical symptoms (6 m–2 y); defects were filled with fibrocartilage (1 and 2 y)
Kuroda et al., 2007 (Japan) [49]	Case report	Full-thickness articular-cartilage defects in medial femoral condyle (ICRS IV). Size: 20 × 30 mm	1 (age 31 y)	1 y	Transplantation of 5 × 10^6^ expanded BMSC per mL collagen gel + periosteum	Clinical symptoms were improved; histology revealed defect filling with 3 layers of repair tissue appearing hyaline-like (middle), fibrous (top), and bone-like (lower). MRI revealed tissue irregularities
Wakitani et al., 2007 (Japan) [50]	Case report	Full-thickness articular-cartilage defects in patella femoral joints. Size: 1: 1.6 and 1*.*0 cm^2^ 2: 4*.*2 cm^2^ 3: 1.1 and 1*.*0 cm^2^	3 (age 31 y, 44 y, and 45 y)	17–27 m	Transplantation of 5 × 10^6^ expanded BMSC per mL collagen gel + periosteum or synovium	Clinical symptoms were improved; in 2 patients defect was filled with fibrocartilaginous tissue
Haleem et al., 2010 (Egypt) [51]	Case series	Full-thickness articular-cartilage defects in medial femoral condyle. Size: 3 to 12 cm^2^ (Outerbridge III/ IV)	5 ( age 21–37 y)	12–16 m	2 × 10^6^ cells per cm^2^ expanded BMSC + 3 mL PRF	Improved clinical scores; MRI revealed partial defect filling with complete or incomplete congruity for 2 patients, and for 3 patients complete defect filling without hypertrophy and with congruity of repair tissue with native cartilage
Nejadnik et al., 2010 (Singapore) [52]	Comparative study	Full-thickness cartilage defects in knees	72 total (age < 65 y): 36 ACI; 36 BMSC	Up to 24 m	Transplantation of chondrocytes or 2 × 10^6^ BMSC + periosteum	Overall no difference in clinical scores between ACI and BMSC groups; men had greater improvement than women; histology of biopsies from 7 patients revealed hyaline-like cartilage
Varma et al., 2010 (India) [53]	Comparative study	Mild to moderate knee OA	50 total: 25 BMC + debridement; 25 debridement	?	Injection of BMC concentrate	Improvement of visual analogue scale (VAS) score, OA outcome score, and quality of life
Kim et al., 2014 (South Korea) [54•]	Case series	Radiological degree of OA; there were 12, 24, 33, and 6 cases of each of Kellgren–Lawrence grade I, II, III, and IV	41 (age 53–80 y)	3, 6, 12 m	Injection of BMC concentrate + adipose tissue	BMC injection significantly improved both knee pain (VAS score) and function. VAS score improvement was least in the K–L grade IV group
Centeno et al., 2008 (USA) [55]	Case report	MRI evidence of degenerative knee OA	1 (age 46 y)	6 m	Injection of 22.4 × 10^6^ expanded BMSC + 1 mL PRP	Femoral cartilage volume increase determined from MRI, functional scores improvement (VAS and FRI), and increase in range of motion in extension
Centeno et al., 2010 (USA) [56]	Case series	Degenerative joint and disc diseases	213 (mean age 52.8 ± 13.5 y)	3, 6, 12, 24 m	Injection of 1–3 × 10^6^ expanded BMSC + 10–20 % PRP	No evidence of neoplastic complications in any re-implant site by MRI
Centeno et al., 2011 (USA) [57]	Case series	Degenerative joint and disc diseases	340 (53 ± 13.85 y)	3, 6, 12, 24 m	Injection of expanded BMSC + PRP	Knee outcome scores: >75 % improvement was reported for 41.4 % while reducing the improvement threshold to >50 % improvement, 63.2 % reported an improvement; no evidence of neoplastic complications in any re-implant site on MRI
Emadedin et al., 2012 (Iran) [58•]	Case series	Radiological evidence of knee OA	6 (mean age 54.5 y)	12 m	Injection of 20–24 × 10^6^ expanded BMSC	Pain, functional status, and walking distance improved; MRI indicated increase in cartilage thickness and extension of the repair tissue over the SB in 3/6 patients
Davatchi et al. 2011 (Iran) [59]	Case report	Moderate to severe knee OA	4 (age 55, 57, 65, and 54 y)	6 m	Injection of 8–9 × 10^6^ expanded BMSC	Mild improvement of pain on the basis of walking time, stair climbing, and VAS
Pak, 2011 (South Korea) [60•]	Case report	Knee OA	2 (age 70 y and 79 y)	12 weeks	Injection of ADSC (not expanded) + PRP + HA + dexamethasone	Improvement of pain and range of motion; increase of height and thickness of meniscus cartilage on the medial side
Koh et al., 2012 (South Korea) [61]	Comparative study	Knee OA	50 total: 25 ADSC + PRP; 25 PRP	16 m	Injection of 1.9 × 10^6^ concentrated ADSC after debridement (not expanded) + 3 mL PRP	Mean Lysholm, Tegner activity scale, and VAS scores improved significantly, but no significant differences between study and control groups
Koh et al., 2013 (South Korea) [62]	Case series	Knee OA	18 (mean age 55 y)	26 m	Injection of 03–2.7 × 10^6^ concentrated ADSC (increasing number) after debridement (not expanded) + 3 mL PRP	Improvements in clinical scores (VAS, OA indices, Lysholm) and MRI results were positively related to the number of stem cells injected
Koh et al., 2013 (South Korea) [63]	Case series	Knee OA	30 (age > 65 y)	3, 12 and 24 m	Injection of 4.2 × 10^7^ expanded ADSC + 3 mL PRP	25/30 patients had improved KOOS, Lysholm, and VAS scores
Wakitani et al., 2002 (Japan) [64]	Comparative study	Severe knee OA	24 total (mean age: 63 y range: 49-70 y): 12 BMSC + collagen gel + collagen sheet + periosteum; 12 cell-free controls	42 weeks	Implantation of 1.3 × 10^7^ expanded BMSC + collagen gel + collagen sheet	Defects were coved with soft white cartilage-like tissue; Although the clinical improvement was not significantly different, the arthroscopic and histological grading score was better in the cell-transplanted group than in the cell-free control group
Koh et al., 2014 (South Korea) [65•]	Case series	Isolated full-thickness articular-cartilage lesions because of knee OA. Size: 5.4–2.9 cm^2^	35 (mean age 57.4 y, range 48–69 y)	24–34 m	Implantation of 3.8 × 10^6^ ADSC (not expanded)	Clinical score (IKDC and Tegner activity scale) improved; on the basis of ICRS repair score, 2 lesions were normal, 7 near normal, 20 abnormal, and 8 severely abnormal: 76 % of repair was rated as abnormal or severely abnormal
Wong et al., 2013 (Singapore) [66]	Comparative study	Uni-compartmental knee OA	56 total (mean age 51 y): 28 MFX + BMSC; 28 MFX	12 m	Injection of 1.5 × 10^7^ expanded BMSC after MFX	Improvement in clinical scores (Tegner, IKDC, Lysholm) but significantly better in BMSC group; MRI revealed significantly better MOCART scores for BMSC group

ACI = autologous chondrocyte implantation, ADSC = adipose-derived mesenchymal stem cells, AMIC = autologous matrix-induced chondrogenesis, BMC = bone-marrow concentrate, BMSC = bone-marrow-derived mesenchymal stem cells, FRI = functional rating index, HA = hyaluronic acid, ICRS = International Cartilage Repair Society, IKDC = International Knee Documentation Committee, KOOS = osteoarthritis outcome score, m = months, MFX = microfracture, MOCART = magnetic-resonance observation of cartilage repair tissue, MRI = magnetic-resonance imaging, OA = osteoarthritis, PGA–HA = polyglycolic acid–hyaluronan, PRF = platelet-rich fibrin gel (glue), PRP = platelet-rich plasma, SB = subchondral bone, VAS = visual analogue scale, WOMAC = Western Ontario and McMaster Universities Osteoarthritis Index, y = years.

##### Application of Expanded BMSC to Trauma-Induced Defects

The second principal treatment strategy, using expanded BMSC, might be difficult to manage from a legal perspective (because it is regarded as pharmacological drug administration), but positive results have been reported from preclinical animal and clinical human studies. The advantage of expanded MSC is a higher number of cells; the disadvantage is an increased contamination risk during expansion [41•]. The Wakitani group was the first to transplant autologous BMSC, aspirated from the iliac crest and embedded in a collagen gel; these were transplanted into full-thickness patella-femoral-articular-cartilage defects of two patients and covered with a periosteal flap. Although clinical symptoms were improved, histological evaluation 12 months later revealed that the defects were filled with fibrocartilage and not with hyaline-like-cartilage tissue [48]. Three years later the group used the same procedure to implant BMSC into full-thickness cartilage defects of patella-femoral joints. Again, clinical symptoms improved and were maintained throughout the follow-up period. However, it was not possible to unambiguously determine whether the defects were repaired with hyaline cartilage, although biopsies were safranin-O or toluidine-blue positive. Much more likely was that the defects were filled with fibrocartilage-like tissue [49, 50]. In 2010, Haleem et al. transplanted BMSC embedded in platelet-rich fibrin glue (PRF) into full-thickness cartilage defects; compared with the collagen sheet (bovine and porcine origin) PRF has the advantages of being autologous and bio-resorbable. On the basis of MRI, after 12 months the authors concluded that in three out of five patients the defects were filled with tissue resembling native cartilage without signs of hypertrophy [51]. The only comparative study, reported by Nejadnik et al., compared transplantation outcomes for autologous collagen-embedded BMSC versus chondroctyes transplanted into full-thickness knee-cartilage defects in a cohort study. Analysis after an up-to-two-years follow-up period revealed similar functional and clinical results, with the clear advantage that the BMSC treatment required only one operation and minimized donor-site morbidity [52].

These cartilage-repair techniques were able to generate repair tissue which up to a certain point approximates the characteristics of naive hyaline cartilage, but which still more closely represents undesired fibrocartilage tissue. MSC and BMC treatment strategies obtained a marked reduction in procedure, morbidity, and cost by using a “one step” technique able to overcome all the disadvantages of previous repair techniques. However, clinical data on both strategies (i.e. delivering cultured MSC or BMC to the defect site), although promising, must be regarded as preliminary because most reported studies were single or series case studies without valid and sound controls (Table 2).

#### OA-Induced Defects

The above intervention strategies were performed mostly on younger patients without clinical and radiological symptoms of OA. Important for the choice of cell-application strategies is the fact that OA is a chronic disease which results in multiple cartilage lesions during pathogenesis, whereas trauma in general results in a single localized chondral or osteochondral defect. Therefore, the easiest way to treat OA-mediated lesions would be intraarticular injection of MSC or BMC.

##### Injection of BMC

In a study by Varma et al., 50 patients with mild to moderate knee OA were selected and divided into two groups. One group received arthroscopic debridement alone and the other received buffy coat (mesenchymal-stem-cell concentrate) injection with the arthroscopic debridement. On follow-up, patients were assessed on the basis of visual analogue scale (VAS) score and OA outcome score. The results suggest that injection of BMC much improved the overall OA outcome score, especially the quality of life within and at the end of the follow-up period [53]. A more recent study by Kim et al. used combined intraarticular injection of autologous BMC and adipose tissue (BMAC) into the knees of 41 patients with radiographically assessed OA (assessed on the basis of the Kellgren–Lawrence (K–L) grade I–IV scale). BMAC injection significantly improved both knee pain and function in all patients. The authors suggest that the injection would be even more effective in early to moderate phases of OA [54•] (Table 2).

##### Injection of BMSCs and ADMSCs

There are several case reports in which BMSCs expanded in vitro were injected into OA joints. The approach of Centeno et al. [55] is interesting, because they augmented the single BMSC injection with autologous platelet lysates prepared from platelet-rich-plasma (PRP) aspirates. The pre and post-procedure MRI analysis revealed increased meniscus and cartilage volume, and at three-month follow-up the modified VAS scores decreased by 95 %. Two subsequent uncontrolled studies from the same group using the same treatment procedure were conducted on a large group of patients suffering from OA and other intraarticular pathology [56, 57]. However, only approximately 60 % of the patients had knee improvement scores thereafter. In this line, suboptimal results were reported after a single injection of expanded and partially characterized BMSCs. This study was a phase-one clinical trial which recruited six patients with radiological evidence of knee OA requiring joint-replacement surgery. Pain, functional status of the knee, and walking distance tended to improve up to six months post-injection, after which pain seemed to be slightly increased and the walking ability of the patients slightly decreased. Comparison of MRI at baseline and six months after the stem-cell injection revealed increased cartilage thickness, increased extension of the repair tissue over the subchondral bone, and much-reduced size of subchondral bone-marrow lesions in three out of six patients [58•]. Similar results were obtained in a second uncontrolled pilot study [59].

Of much clinical interest would be the use of ADSCs obtained from lipoaspirates, which offer several advantages over BMSCs when used for cartilage-tissue engineering. ADSCs are more abundant and easily available, and they confer a similar potential to differentiate and to direct formation of cartilage-like tissue. In 2011, Pak et al. [60•] reported good results after treatment of two patients affected by knee OA with the injection of concentrated ADSCs (not expanded or cultured) together with hyaluronic acid (HA), dexamethasone, and PRP. Concentrated ADSCs were obtained by double centrifugation of lipoaspirates and digestion with collagenase. After three months subjective pain and functional status improved, and MRI revealed significantly increased cartilage thickness [60•]. This was followed by three studies from Koh et al. [61], who used concentrated ADSCs derived from the infrapatellar fatpad for treatment of knee OA. First, they presented a case-control study with a total of 25 patients, who each received a single injection of ADSCs and, subsequently, PRP [61]. Clinical results were promising, and the group conducted two further case studies (not controlled) with a similar treatment procedure, but with a different source of ADSCs in the second study. All applied clinical outcome and MRI cartilage scores improved significantly [62, 63]. These studies suggest that ADSC therapy for knee OA is effective in promoting cartilage healing, reducing pain, and improving function, and therefore seems to be a promising option for OA treatment in elderly patients (Table 2).

##### Implantation of BMSCs and ADSCs

The first report describing the implantation of expanded BMSCs into OA knee lesions also came from the Wakitani group. They conducted a comparative study on patients who had undergone high tibial osteotomy (HTO) because of OA. Twelve patients received transplantation with expanded autologous BMSCs, which were embedded in a gel composed of type I collagen and implanted as a collagen sheet, and 12 patients served as cell-free controls receiving the collagen sheet alone. The BMSC–collagen construct was positioned into a large defect of the medial femoral condyle, where cartilage was lost and subchondral bone was exposed, and covered with a periosteal flap. Approximately a year later clinical scores did not differ between the groups, but arthroscopy and histological scores were better in the cell-transplanted group [64]. In a recent report from the Koh group, ADSCs were implanted into an isolated full-thickness cartilage lesion in OA knees. Patients with multiple cartilage lesions were excluded, as were those with a history of marrow-stimulation procedures, i.e. MFX or subchondral drilling. Directly after isolation from fat tissue, cells were dripped into the cartilage lesion and the knee was held in a stationary position for 10 min. No supporting biomaterial was used, nor were the cells kept in position by covering with a matrix or periosteal flap, but simple adherence to the subchondral bone was permitted. Although the authors suggested that MSC implantation could have great potential for treating OA lesions, the second-look arthroscopy findings revealed that in 76 % of the patients the repair was abnormal or severely abnormal by ICRS standards. They concluded that the development of an advanced surgical procedure with tissue-engineered scaffolds may be needed to treat patients with OA-induced large cartilage lesions [65•].

In general, it is not recommended that the MFX technique alone should be used for treatment of OA-mediated degenerative cartilage lesions. However, in a recent randomized, controlled clinical study, MFX combined with intraarticular injection of BMSCs was used to treat 28 patients with uni-compartmental OA knees undergoing HTO, whereas the second group of 28 patients received an intraarticular HA injection instead. Assessment of IKDC and MOCART scores up to two years post-surgery revealed significantly higher scores in the cell-treated group [66].

##### Concerns Regarding Current Clinical-Treatment Procedures

There are several common limitations, of which at least one applies to each clinical study listed. First and most importantly, most studies lack quantitative and histological evidence. Second, many studies are level IV studies, and therefore either no matched control group is included or the number of cases is very small. In addition, the follow-up period is often too short and it would be desirable to have more than one arthroscopy observation during the follow-up time. The latter, of course, is associated with ethical concerns. Third, almost every study used different treatment procedures, i.e. intraarticular injection versus implantation or including MSCs from different tissue sources, and thus they can be regarded only as pilot studies.

## Conclusions

Only a few published pre-clinical, large animal, and clinical studies of MSC-based treatment of OA-induced chondral and osteochondral lesions are available (Fig. 1). Most pre-clinical studies using large-animal models provide MSC-based treatment procedures for isolated focal chondral and osteochondral lesions encompassed by healthy cartilage and bone. Furthermore, they do not address special requirements for cell-based strategies adapted to treat OA-induced degenerative large cartilage defects. In addition, all studies used expanded BMSCs, requiring a two-step treatment, and none used BMC, which have important clinical implications because they can be applied without leaving the operating theatre, circumventing regulatory obstacles. Clinical studies are mostly uncontrolled case reports including only a few patients, and detailed molecular and histological analyses of repair tissue are not feasible for ethical reasons. In addition, study procedures, follow-up times, cell sources, and biomaterials differ greatly among the studies, thereby preventing generalized conclusions on clinical and functional outcomes. However, preliminary results of pre-clinical and clinical studies are promising. In general, after cell-based therapy—irrespective of cell type—clinical and functional scores are clearly improved and defects are filled with newly formed cartilage-like tissue, sometimes even with hyaline-like characteristics.
